# Supplementary material for: Non-Clinical Safety of GRAd Vector-Based COVID-19 and HIV Vaccines Supports a Platform Regulatory Approach
Source: Vaccines (Basel). 2026 Feb 6;14(2):157. doi: 10.3390/vaccines14020157 (PMC12944892; doi:10.3390/vaccines14020157)
Supplement: Supplementary file 1 [file vaccines-14-00157-s001.zip › vaccines-4117374-supplementary.pdf]

Supplementary Materials

Paalangara R. et al

Table S1: GRAd toxicity and local tolerance studies in New Zealand White Rabbits

	**GRAd-COV2 Single Dose**	**GRAd-COV2 Repeated Dose**	**GRAdHIVNE1 Repeated Dose**
**age (weeks)**<br>**weight mean (sex, kg)**	15-16w<br>M 3.1/F 3.3	13-18w<br>M 3.1/F 2.8	11w<br>M 2.2/F 2.1
**N injections**<br>**interval**	N=1 (d1)	N=2<br>2 weeks (d1; d15)	N=2<br>3 weeks (d1; d22)
**vaccine dose**	1 x 10^{11} vp (500 μL)		
**follow up**	1 week	4 weeks post 2nd injection	
**group numerosity**	5M+5F receiving vaccine or saline/euthanasia time point		
**euthanasia time points**	d8 (7d post admin)	d18 (3d post 2nd)<br>d43 (4w post 2nd)	d25 (3d post 2nd)<br>d49F/d50M (4w post 2nd)
**vaccine take demonstration**	none	Spike Antibodies<br>(serum, ELISA)	T cells to HIV epitopes<br>(PBMC, IFNγ ELISpot)
**survival check**	Once in pretreatment, then twice daily		
**clinical observation**	cageside: daily<br>detailed: once pre, d1@4h, d4, d7	cageside: daily<br>detailed: once pre, d1@4h, then weekly	cageside: daily (on d1 and d22 @0.5h,3h, 8h)<br>detailed: d1 and d22@1h-6h,24h, then weekly
**body temperature**	pre (admin time/4h),<br>@admin(s), 4h, 24h<br>If >40°C daily until resolution		5x pre, d1-d22 @admin, 6h, 24h, 48h, 72h<br>If >40°C daily until resolution (2 occasions)
**body weight**	Thrice pre, admin days (d1) then daily until EOS	Thrice pre, admin days (d1 and d15), daily for next 3 days, then weekly	Once pre, d1, 2, 3, 8, 11, 15, 18, 21, 23, 24, then twice weekly.
**food consumption**	Once pre, d1 then daily	Once pre, d1, then twice weekly	d1, then daily
**local tolerance**	Draize score: @admin(s), 4h, 24h, 48h,72h		Draize score: @admin, 6h, 24h, 48h,72h, EOS
**ophthalmology**	none	once pre, EOS	
**clinical pathology (hematology, biochemistry, coagulation, CRP)**	pre, d1(pre), d2, d4, d8 (EOS)	pre, @admin(pre), d2, d4, d8 after each admin, d43 (EOS)	pre, d2 (CRP only), d4, d25/d49 (EOS)
**anatomic pathology**	necropsy<br>organ weight,<br>preservation (limited list)<br>histopathology of inj. site and draining LN	necropsy<br>organ weight (selected list), preservation, histopathology (full WHO list)	

Supplementary table 1 abbreviations: h=hour; d=day; w=week; pre=pre-dose; admin=administration; EOS=end of study

Table S2: GRAd-COV2 Biodistribution Results (Genome Copies/µg of DNA)

Organ	Sex	Day 2			Day 8			Day 30			Day 49		
		No. of samples (/5)			No. of samples (/5)			No. of samples (/5)			No. of samples (/5)		
		BLD[a]	BLQ[b]	Quantified[c]	BLD[a]	BLQ[b]	Quantified[c]	BLD[a]	BLQ[b]	Quantified[c]	BLD[a]	BLQ[b]	Quantified[c]
Blood	F	0	4	1<br>3.61E+02	5	0	0						
	M	2	3	0	5	0	0						
Right Iliac LN	F	0	0	5<br>Mean : 1.32E+04<br>(1.85E+03 - 3.99E+04)	0	0	5<br>Mean : 1.23E+02<br>(8.20E+01 - 1.89E+02)	0	3	2<br>Mean : 5.65E+01<br>(5.56E+01 - 5.74E+01)	0	1	4<br>Mean : 1.24E+03<br>(7.96E+02 - 1.65E+03)
	M	1	0	4<br>Mean : 1.55E+04<br>(1.99E+03 - 2.63E+04)	0	0	5<br>Mean : 2.24E+03<br>(9.71E+01 - 5.45E+03)	5	0	0	1	0	4<br>Mean : 1.16E+03<br>(8.98E+02 - 1.47E+03)
Right Inguinal LN	F	3	0	2<br>Mean : 1.16E+03<br>(2.86E+02 - 2.02E+03)	3	2	0	5	0	0	1	2	2<br>Mean : 8.26E+02<br>(6.60E+01 - 1.59E+03)
	M	1	1	3<br>Mean : 2.71E+03<br>(4.98E+02 – 6.84E+03)	1	1	3<br>Mean : 3.33E+02<br>(1.21E+02 - 7.27E+02)	4	1	0	2	1	2<br>Mean : 1.53E+02<br>(1.00E+02 - 2.05E+02)
Quadriceps muscle	F	0	0	5<br>Mean : 9.76E+04<br>(7.34E+02 - 3.84E+05)	0	1	4<br>Mean : 6.69E+02<br>(9.38E+01 - 1.72E+03)	0	0	5<br>Mean : 2.01E+02<br>(6.71E+01 - 4.63E+02)	1	0	4<br>Mean : 5.82E+02<br>(8.93E+01 - 1.82E+03)
	M	0	0	5<br>Mean : 1.15E+04<br>(7.53E+01 - 3.76E+04)	0	0	5<br>Mean : 5.83E+03<br>(9.51E+01 - 1.13+04)	2	1	2<br>Mean : 7.84E+01<br>(6.64E+01 - 9.05E+01)	1	0	4<br>Mean : 6.24E+02<br>(9.77E+01 - 2.10E+03)

[a] Below limit of detection (i.e., < 6.25 genome copies/well).
[b] Below limit of quantification (i.e., < 50 genome copies/well).
[c] Number of quantified samples and mean of test item DNA quantity (genome copies/µg of DNA) when applicable (i.e., unless only one sample returned a quantified value). In brackets: Min and Max values.

In grey = Analysis not performed as no test item genomic DNA was quantified at the two previous time points. LN = Lymph Nodes. No = Number

Table S3: GRAdHIVNE1 Biodistribution Results (Genome Copies/µg of DNA)

Organ	Sex	Day 2			Day 8			Day 29			Day 49		
		No. of Samples (/5)			No. of Samples (/5)			No. of Samples (/5)			No. of Samples (/5)		
		BLD[a]	BLQ[b]	Quantified[c]	BLD[a]	BLQ[b]	Quantified[c]	BLD[a]	BLQ[b]	Quantified[c]	BLD[a]	BLQ[b]	Quantified[c]
Blood	F	0	1	4<br>Mean: 1.99E+03<br>(3.03E+02-3.62E+03)	5	0	0	4	1	0			
	M	0	2	3<br>Mean: 1.56E+02<br>(8.03E+01-3.03E+02)	5	0	0	5	0	0			
Iliac LN	F	0	1	4<br>Mean: 9.78E+04<br>(6.21E+04-1.17E+05)	0	0	5<br>Mean: 5.87E+03<br>(4.18E+03-7.15E+03)	0	0	5<br>Mean: 5.09E+03<br>(3.75E+03-7.01E+03)	1	0	4<br>Mean: 4.67E+03 (2.96E+03-6.35E+03)
	M	0	1	4<br>Mean: 6.68E+04,<br>(5.33E+04-7.88E+04)	0	0	5<br>Mean: 4.96E+03<br>(3.69E+03-7.33E+03)	0	1	4<br>Mean: 2.88E+03<br>(1.90E+03-4.33E+03)	1	1	3<br>Mean: 2.70E+03 (1.52E+03-3.47E+03)
Inguinal LN	F	1	1	3<br>Mean: 1.53E+04<br>(1.64E+02-4.55E+04)	0	1	4<br>Mean: 9.74E+02<br>(7.24E+01-1.91E+03)	0	2	3<br>Mean:1.32E+02<br>(5.99E+01-2.67E+02)	1	3	1<br>4.56E+03
	M	1	2	2<br>Mean:1.88E+04<br>(7.68E+01-3.74E+04)	0	3	2<br>Mean:1.71E+03<br>(7.37E+01-3.34E+03)	2	3	0	1	1	3<br>Mean: 1.32E+03 (1.01E+02-3.21E+03)
Quadriceps muscle	F	0	0	5<br>Mean: 5.10E+06<br>(5.86E+05-1.43E+07)	0	0	5<br>Mean: 5.00E+04<br>(9.34E+03-7.90E+04)	0	0	5<br>Mean: 1.04E+04<br>(3.52E+02-1.42E+04)	0	0	5<br>Mean: 7.27E+03 (2.08E+02-1.78E+04)
	M	0	0	5<br>Mean: 2.08E+06<br>(5.04E+05-7.04E+06)	0	0	5<br>Mean: 2.35E+04<br>(4.90E+02-4.60E+04)	0	0	5<br>Mean: 4.44E+03<br>(3.04E+02-7.53E+03)	0	0	5<br>Mean: 4.28E+03<br>(3.85E+02-1.02E+04)
Spleen	F	0	2	3<br>Mean:1.96E+02<br>(5.04E+01-3.19E+02)	0	5	0	0	2	3<br>Mean:9.79E+01<br>(6.72E+01-1.29E+02)	0	3	2<br>Mean:1.26E+02 (1.22E+02-1.31E+02)
	M	2	3	0	1	4	0	4	1	0	1	4	0

[a] Below limit of detection (i.e., < 6.25 genome copies/well).
[b] Below limit of quantification (i.e., < 50 genome copies/well).
[c] Number of quantified samples and mean of test item DNA quantity (genome copies/µg of DNA) when applicable (i.e., unless only one sample returned a quantified value). In brackets: Min and Max values.

In grey = Analysis not performed as no test item genomic DNA was quantified at the two previous time points. LN = Lymph Nodes. No = Number

Table S4: GRAd-COV2 RD toxicity study body temperature (°C)

Sex	Male		Female	
Group	1	2	1	2
Treatment	Control (Saline)	Vaccine (GRAd-COV2)	Control (Saline)	Vaccine (GRAd-COV2)
1st administration: Day 1				
. Day 1 (pre-dose)	39.0	38.9	39.1	39.1
. Day 1 (4 hours)	38.6	38.9	39.1	39.1
. Day 2 (24 hours)	38.5	39.0**	38.6	**39.5****
. Day 3 (48 hours)	-	-	-	38.8 (n = 3)
2nd administration: Day 15				
. Day 15 (pre-dose)	39.3	39.3	39.1	38.9
. Day 15 (4 hours)	38.7	38.7	38.8	39.1
. Day 16 (24 hours)	38.7	38.9	38.8	38.7

Statistically significant differences from controls: ** ($p<0.01$).

Bold values: considered as test item-related. -: not applicable; n: number of animals.

Table S5: GRAd-COV2 RD toxicity study summary of Hematology Changes Compared to Control (Group 1) Values

Parameter/ Study Day	Males		Females	
	Group 1	Group 2	Group 1	Group 2
	0 vp/animal	1 x 10^{11} vp/animal	0 vp/animal	1 x 10^{11} vp/animal
**White Blood cells (G/L)**				
Pretest	5.36	1.00x	6.59	1.04x
Day 16	4.85	**1.27x***	5.44	**1.49x****
Day 18	4.91	**1.22x**	7.36	**1.11x**
**Neutrophils (G/L)**				
Pretest	1.13	1.09x	1.22	1.18x
Day 2	0.83	**1.64***	1.28	**1.93x**
Day 16	0.78	**2.20x****	0.95	**2.70x*****
**Lymphocytes (G/L)**				
Pretest	3.62	0.93x	4.55	1.02x
Day 2	3.55	**0.83x***	3.84	**0.68x***
**Monocytes (G/L)**				
Pretest	0.11	1.27x	0.15	0.73x
Day 4	0.11	1.55x	0.19	**2.16x**
Day 18	0.08	2.13x	0.25	**1.68x**
**Eosinophils (G/L)**				
Pretest	0.09	1.44x	0.13	0.77x
Day 2	0.12	**0.50x***	0.08	**0.38x****
**Basophils (G/L)**				
Pretest	0.39	1.10x	0.51	1.06x
Day 2	0.41	**0.80x**	0.46	**0.76x**
**Large unstained cells (G/L)**				
Pretest	0.03	0.67x	0.03	0.67x
Day 16	0.02	1.00x	0.03	**1.67x**
Day 18	0.02	**2.50x**	0.03	**2.33x**
**Platelets (G/L)**				
Pretest	386	0.83x	397	1.04x
Day 2	410	**0.79x**	406	**0.76x**

Vp = viral particle, Statistically significant from controls: * ($p<0.05$); ** ($p<0.01$); *** ($p<0.001$).
Bold values: considered as test item-related;
The fold change (x) of test item-related findings relative to the control values are listed. For comparison, the control (Group 1) are listed.

Table S6: GRAd-COV2 RD toxicity study summary of Coagulation Changes Compared to Control (Group 1) Values

Parameter/ Study Day	Males		Females	
	Group 1	Group 2	Group 1	Group 2
	0 vp/animal	1 x 10^{11} vp/animal	0 vp/animal	1 x 10^{11} vp/animal
**Fibrinogen (g/L)**				
Pretest	2.85	0.98x	2.02	0.91x
Day 2	2.96	**1.43x****	1.99	**1.97x*****
Day 4	2.81	**1.35x****	1.87	**1.85x*****
Day 16	2.58	**1.51x*****	1.93	**2.02*****
Day 18	2.65	**1.23x****	1.91	**1.48***

Vp = viral particle, Statistically significant from controls: * (p<0.05); ** (p<0.01); *** (p<0.001).
Bold values: considered as test item-related;
The fold change (x) of test item-related findings relative to the control values are listed. For comparison, the control (Group 1) are listed.

Table S7: GRAd-COV2 RD toxicity study summary of Blood Biochemistry Changes Compared to Control (Group 1) Values

Parameter/ Study Day	Males		Females	
	Group 1	Group 2	Group 1	Group 2
	0 vp/animal	1 x 10^{11} vp/animal	0 vp/animal	1 x 10^{11} vp/animal
**Albumin (g/L)**				
Pretest	45	0.97x	41	1.05x
Day 16	44	1.00x	40	1.05x
Day 22	44	1.02x	41	1.02x
**A/G**				
Pretest	3.41	0.92	3.58	1.06
Day 16	3.44	**0.87x***	3.60	**0.92x***
Day 22	3.50	**0.87x****	4.00	0.90x

Vp = viral particle, Statistically significant from controls: * (p<0.05); ** (p<0.01); *** (p<0.001).
Bold values: considered as test item-related;
The fold change (x) of test item-related findings relative to the control values are listed. For comparison, the control (Group 1) are listed.

Table S8: GRAd-COV2 RD toxicity study summary of C-Reactive Protein (CRP) Levels Changes Compared to Control (Group 1) Values

Parameter/ Study Day	Males		Females	
	Group 1	Group 2	Group 1	Group 2
	0 vp/animal	1 x 10^{11} vp/animal	0 vp/animal	1 x 10^{11} vp/animal
**CRP (µg/mL)**				
Pretest	2.24	2.41x	5.93	1.12x
Day 2	4.10	**23.31x*****	7.44	**12.01*****
Day 16	1.86	**37.19x***	9.79	**10.50***

Vp = viral particle, Statistically significant from controls: * (p<0.05); ** (p<0.01); *** (p<0.001).
Bold values: considered as test item-related;
The fold change (x) of test item-related findings relative to the control values are listed. For comparison, the control (Group 1) are listed.

Table S9: GRAd-COV2 RD toxicity study summary of Treatment-related Findings in Mean Organ Weights Compared to Control (Group 1) Values at Early Euthanasia

	Male<br>Dosage (vp/animal/adm.)	Female<br>Dosage (vp/animal/adm.)
	**1×10^{11}**	**1×10^{11}**
**Spleen**		
.absolute	+42	+48*
.relative to body weight	+52	+48*
.relative to brain weight	+53	+55*
**Right iliac lymph node**		
.absolute	+165	+194*
.relative to body weight	+178	+194*
.relative to brain weight	+184	+210*
**Right popliteal lymph node**		
.absolute	+65**	+67
.relative to body weight	+79**	+63
.relative to brain weight	+77**	+77
**Right inguinal lymph node**		
.absolute	+63	-13
.relative to body weight	+70	-12
.relative to brain weight	+68	-8
**Adrenal glands**		
.absolute	+23	+14
.relative to body weight	+31*	+15
.relative to brain weight	+32*	+21*
**Thymus**		
.absolute	-25	-14
.relative to body	-19	-16
.relative to brain	-21	-9

Statistically significant from controls: *: $p<0.05$, **: $p<0.01$.

The significance concerned the organ weights values and not the percentages.

Table S10: GRAd-COV2 RD toxicity study summary of Treatment-related Findings in Mean Organ Weights Compared to Control (Group 1) Values at Late Euthanasia

	Male Dosage (vp/animal/adm.)	Female Dosage (vp/animal/adm.)
	**1×10^{11}**	**1×10^{11}**
**Right iliac lymph node**		
.absolute	+118*	-30
.relative to body weight	+97*	-25
.relative to brain weight	+125*	-29
**Right popliteal lymph node**		
.absolute	+26*	+28
.relative to body weight	+15	+33
.relative to brain weight	+28*	+26
**Left iliac lymph node**		
.absolute	+58	-8
.relative to body weight	+46	+6
.relative to brain weight	+62	-8

Statistically significant from controls: *: $p<0.05$

The significance concerned the organ weights values and not the percentages.

Table S11: GRAd-COV2 RD toxicity study summary of Treatment-related Macroscopic Findings Compared to Control (Group 1) Values at Early Euthanasia

**Finding**	**Male** Dosage (vp/animal/adm.)		**Female** Dosage (vp/animal/adm.)	
	**0**	**1×10^{11}**	**0**	**1×10^{11}**
**Right iliac lymph node** (number examined)	5	5	5	5
Enlarged	-	2	-	4
**Left iliac lymph node** (number examined)	5	5	5	5
Enlarged	-	-	-	1

-: finding not present.

Table S12: GRAd-COV2 RD toxicity study summary of Treatment-related Microscopic Findings Compared to Control (Group 1) Values at Early Euthanasia

Finding	Male Dosage (vp/animal/adm.)		Female Dosage (vp/animal/adm.)	
	0	1×10^{11}	0	1×10^{11}
**Spleen** (*number examined*)	*5*	*5*	*5*	*5*
Cellularity: increased; lymphoid				
Minimal (grade 1)	-	3	-	1
Slight (grade 2)	-	2	-	2
Moderate (grade 3)	-	-	-	2
**Right iliac lymph node** (*number examined*)	*5*	*4*	*5*	*5*
Cellularity: increased; lymphoid				
Slight (grade 2)	-	2	-	-
Moderate (grade 3)	-	2	-	2
Marked (grade 4)	-	-	-	3
Intrasinusoidal erythrocytes				
Minimal (grade 1)	-	3	1	2
**Right inguinal lymph node** (*number examined*)	*5*	*4*	*4*	*5*
Cellularity: increased; lymphoid				
Minimal (grade 1)	-	1	-	1
Intrasinusoidal erythrocytes				
Minimal (grade 1)	-	-	1	-
**Right popliteal lymph node** (*number examined*)	*5*	*5*	*5*	*5*
Cellularity: increased; lymphoid				
Minimal (grade 1)	-	2	-	-
Slight (grade 2)	-	1	-	4
Erythrophagocytosis				
Minimal (grade 1)	-	-	-	1
**Left iliac lymph node** (*number examined*)	*4*	*5*	*5*	*5*
Cellularity: increased; lymphoid				
Minimal (grade 1)	-	1	-	-
Slight (grade 2)	-	1	-	-
Moderate (grade 3)	-	2	-	1
Intrasinusoidal erythrocytes				

Minimal (grade 1)	-	-	-	1
Slight (grade 2)	-	1	-	1
**Left inguinal lymph node** (*number examined*)	*5*	*4*	*4*	*4*
Cellularity: increased; lymphoid				
Minimal (grade 1)	-	-	-	1
**Left popliteal lymph node** (*number examined*)	*5*	*5*	*5*	*5*
Cellularity: increased; lymphoid				
Minimal (grade 1)	-	-	-	2
**Injection site 2** (*number examined*)	*5*	*5*	*5*	*5*
Infiltrate; mononuclear inflammatory cell infiltrate				
Minimal (grade 1)	-	3	-	2
Infiltrate; mixed inflammatory cell infiltrate				
Minimal (grade 1)	-	1	-	-
Slight (grade 2)	-	-	-	1
**Sciatic nerve** (*number examined*)	*5*	*5*	*4*	*5*
Infiltrate; mononuclear inflammatory cell infiltrate				
Minimal (grade 1)	-	1	-	-
Slight (grade 2)	-	1	-	4
Infiltrate; mixed inflammatory cell infiltrate				
Minimal (grade 1)	-	1	-	-
Slight (grade 2)	-	1	-	-
**Femur** (*number examined*)	*5*	*5*	*5*	*5*
Infiltrate; mixed inflammatory cell infiltrate				
Minimal (grade 1)	-	-	-	1

-: finding not present.

Table S13: GRAd-COV2 RD toxicity study summary of Treatment-related Microscopic Findings Compared to Control (Group 1) Values at Late Euthanasia

Finding	Male Dosage (vp/animal/adm.)		Female Dosage (vp/animal/adm.)	
	**0**	**1 x 10^{11}**	**0**	**1 x 10^{11}**
**Spleen** (*number examined*)	*5*	*5*	*5*	*5*
Cellularity: increased; lymphoid				
Minimal (grade 1)	-	4	-	4
**Right iliac lymph node** (*number examined*)	*5*	*5*	*5*	*5*
Cellularity: increased; lymphoid				
Slight (grade 2)	-	2	-	4
Moderate (grade 3)	-	3	-	-
**Right popliteal lymph node** (*number examined*)	*5*	*5*	*5*	*5*
Cellularity: increased; lymphoid				
Minimal (grade 1)	-	1	-	4
Slight (grade 2)	-	3	-	-
**Left iliac lymph node** (*number examined*)	*5*	*4*	*5*	*5*
Cellularity: increased; lymphoid				
Minimal (grade 1)	-	-	1	-
Slight (grade 2)	-	-	-	1
Moderate (grade 3)	-	2	-	2
**Left popliteal lymph node** (*number examined*)	*5*	*5*	*5*	*5*
Cellularity: increased; lymphoid				
Minimal (grade 1)	-	-	-	3

-: finding not present.

Table S14: GRAdHIVNE1 RD toxicity study body temperature (°C)

Sex: male	study day	-7 → -1	-6	-5	-4	-3	-2	1 (PD)	1 (6h PE)	2 (24h PE)	3 (48h PE)	4 (72h PE)	22 (PD)	22 (6h PE)	23 (24h PE)	24 (48h PE)	25 (72h PE)
Group 1	Mean	38.2	38.3	38.0	38.2	38.2	38.3	38.3	38.5	38.3	38.5	38.3	38.6	38.9	39.0	39.0	38.3
0	SD	0.2	0.4	0.3	0.3	0.3	0.3	0.5	0.3	0.3	0.4	0.4	0.2	0.2	0.3	0.2	0.5
vp/dose	N	.	8	8	8	8	8	8	8	8	8	8	8	8	8	8	8
Group 2	Mean	38.2	38.3	38.3	38.2	38.0	38.2	38.3	38.6	39.4 *	38.9	38.0	38.5	39.0	38.6	38.7	38.3
1.0E+11	SD	0.4	0.7	0.4	0.4	0.4	0.6	0.3	0.4	0.5	0.6	0.5	0.3	0.4	0.5	0.4	0.6
vp/dose	N	.	9	10	10	10	10	10	10	10	10	10	10	10	10	10	10

Sex: Female	study day	-7 → -1	-7	-6	-5	-4	-3	1 (PD)	1 (6h PE)	2 (24h PE)	3 (48h PE)	4 (72h PE)	22 (PD)	22 (6h PE)	23 (24h PE)	24 48h PE)	25 (72h PE)
Group 1	Mean	38.4	38.5	38.4	38.3	38.5	38.5	38.6	38.7	38.4	38.5	38.3	38.5	38.9	39.3	39.0	38.3
0	SD	0.2	0.5	0.3	0.3	0.3	0.2	0.4	0.2	0.3	0.3	0.4	0.4	0.4	0.2	0.2	0.6
vp/dose	N	.	8	8	8	8	8	8	8	8	8	8	8	8	8	8	8
Group 2	Mean	38.5	38.5	38.6	38.6	38.5	38.5	38.6	38.9	40.3 *	39.1 *	38.8	38.7	39.2	39.1	39.0	38.1
1.0E+11	SD	0.4	0.6	0.5	0.5	0.4	0.4	0.3	0.4	0.5	0.5	0.5	0.3	0.4	0.2	0.2	0.5
vp/dose	N	.	10	10	10	10	10	10	10	10	10	10	10	10	10	10	10

Anova & Dunnett: * = $p < 0.05$

Table S15: GRAdHIVNE1 RD toxicity study summary of Hematology Changes Compared to Control (Group 1) Values

Parameter/ Study Day	Males		Female	
	Group 1	Group 2	Group 1	Group 2
	0 vp/dose	1.0E+11 vp/dose	0 vp/dose	1.0E+11 vp/dose
**Red Blood Cells (× 10^6 cells/µL)**				
Day 4	6.03	**0.9×[a]**	5.57	**0.9×[a]**
Day 25	6.09	**0.9×[a]**	5.41	1.0×
Day 49/50	6.00	1.0×	5.31	1.1×
**Hemoglobin (g/dL)**				
Day 4	12.3	**0.9×[a]**	11.3	**0.9×[a]**
Day 25	12.3	**0.9×[a]**	10.9	1.0×
Day 49/50	12.3	1.0×	11.0	1.0×
**Hematocrit (%)**				
Day 4	37.2	**0.9×[a]**	34.7	**0.9×[a]**
Day 25	37.7	**0.9×[a]**	34.0	1.0×
Day 49/50	37.2	1.0×	33.2	1.1×
**Monocytes (× 10^3 cells/µL)**				
Day 4	0.05	**10.8×[a]**	0.09	**4.7×[a]**
Day 25	0.09	**2.6×[a]**	0.07	**3.3×[a]**
Day 49/50	0.04	0.8×	0.06	0.8×
**Monocytes (%)**				
Day 4	0.65	**11.7×[a]**	1.50	**4.6×[a]**
Day 25	1.43	**2.1×[a]**	1.58	**2.7×[a]**
Day 49/50	0.98	0.7×	1.58	0.9×
**Large Unstained Cells (× 10^3 cells/µL)**				
Day 4	0.01	**3.0×[a]**	0.01	**2.0×[a]**
Day 25	0.01	**2.0×[a]**	0.00	**-[a]**
Day 49/50	0.00	-	0.00	-
**Large Unstained Cells (%)**				
Day 4	0.1	**4.0×[a]**	0.2	**2.0×[a]**
Day 25	0.1	**3.0×[a]**	0.1	**2.0×[a]**
Day 49/50	0.1	1.0×	0.1	1.0×
**Mean Platelet Volume (fL)**				
Day 4	10.3	**1.1×[a]**	9.1	**1.0×[a]**
Day 25	9.1	1.0×	9.1	1.0×
Day 49/50	10.2	1.0×	9.1	1.0×

vp = viral particle, a = $p < 0.05$, - = no calculatable fold change

The fold change (×) of test article-related findings relative to the control values are listed. For comparison, the control (Group 1) values are listed.

Table S16: GRAdHIVNE1 RD toxicity study summary of Serum Chemistry Changes Compared to Control (Group 1) Values

Parameter/ Study Day	Males		Female	
	Group 1	Group 2	Group 1	Group 2
	0 vp/dose	1.0E+11 vp/dose	0 vp/dose	1.0E+11 vp/dose
**C-Reactive Protein (mg/L)**				
Pre-dose	<	<	30.5	<
Day 2	18.0	**4.1×**[a]	21.2	**4.7×**[a]
Day 4	8.7	**7.3×**[a]	18.6	**3.4×**[a]
Day 25	<	-	<	-
Day 49/50	7.6	1.0×	<	-
**Creatine Kinase (U/L)**				
Day 4	1658	>	>	>
Day 25	871	**1.6×**[a]	1172	1.1×
Day 49/50	1069	1.2×	>	-
**Rabbit Globulin (g/dL)**				
Day 4	1.4	**1.1×**[a]	1.3	**1.2×**[a]
Day 25	1.3	**1.2×**[a]	1.1	**1.3×**[a]
Day 49/50	1.3	1.0×	1.2	1.0×
**Hemolysis Index**				
Day 4	7	**0.6×**[a]	4	1.5×
Day 25	14	0.7×	13	0.5×
Day 49/50	11	1.1×	12	0.9×
**Lipemia Index**				
Day 4	7	1.0×	5	1.2×
Day 25	2	1.5×	1	**4.0×**[a]
Day 49/50	1	2.0×	1	2.0×

vp = viral particle; a = $p < 0.05$; - = No calculated data available due to linearity exclusion.
< = Result below instrument linearity limit, too low to enumerate accurately. > = Result above instrument linearity limit, too high to enumerate accurately. The fold change (×) of test article-related findings relative to the control values are listed. For comparison, the control (Group 1) values are listed.

Table S17: GRAdHIVNE1 RD toxicity study summary of Coagulation Changes Compared to Control (Group 1) Values

	Males		Female	
	**Group 1**	**Group 2**	**Group 1**	**Group 2**
	**0 vp/dose**	**1.0E+11 vp/dose**	**0 vp/dose**	**1.0E+11 vp/dose**
**Prothrombin Time (seconds)**				
Day 4	8.8	1.0×	9.0	**0.9×[a]**
Day 25	8.9	**1.0×[a]**	8.9	**0.9×[a]**
Day 49/50	9.0	1.0×	8.6	1.0×
**Activated Partial Thromboplastin Time (seconds)**				
Day 4	14.1	1.0×	13.8	**0.9×[a]**
Day 25	14.7	0.9×	15.6	**0.9×[a]**
Day 49/50	15.1	1.1×	16.5	1.0×
**Fibrinogen (mg/dL)**				
Day 4	355	**1.7×[a]**	301	**1.7×[a]**
Day 25	295	**1.5×[a]**	251	**1.6×[a]**
Day 49/50	275	0.9×	216	1.0×

vp = viral particle, a = $p < 0.05$

The fold change (×) of test article-related findings relative to the control values are listed. For comparison, the control (Group 1) values are listed.

Table S18: GRAdHIVNE1 RD toxicity study Summary of Treatment-Related Findings in the Injection Sites and Skeletal Muscle – Main Cohort (Day 25)

	Males		Females	
**Group**	**1**	**2**	**1**	**2**
**Dose (vp/dose)**	**0**	**1.0E+11**	**0**	**1.0E+11**
**No. of animals examined**	**4**	**5**	**4**	**5**
**Injection site 1 (no. examined)**	4	5	4	5
Inflammation, skeletal muscle, mononuclear cell	0	2 (1.5)	0	1 (2.0)
Degeneration, skeletal muscle	0	1 (1.0)	0	0
**Injection site 2 (no. examined)**	4	5	4	5
Inflammation, skeletal muscle, acute	0	0	0	1 (2.0)
Hemorrhage, skeletal muscle	0	0	0	1 (2.0)
Necrosis, skeletal muscle	0	2 (1.0)	0	1 (2.0)
Regeneration, skeletal muscle	0	0	0	1 (1.0)
Hemorrhage, dermis	0	0	0	1 (2.0)
**Skeletal muscle**	4	5	4	5
Inflammation, mononuclear cell	0	1 (1.0)	0	1 (1.0)
Necrosis	0	0	1 (2.0)	1 (2.0)
Regeneration	0	1 (1.0)	0	0

Average severity (in parentheses) was calculated by adding all the grades of affected animals in the group and dividing by the number affected.

Table S19: GRAdHIVNE1 RD toxicity study Summary of Treatment-Related Findings in the Injection Site 2 and Skeletal Muscle – Recovery Cohort (Day 49/50)

	Males		Females	
**Group**	**1**	**2**	**1**	**2**
**Dose (vp/dose)**	0	1.0E+11	0	1.0E+11
**No. of animals examined**	4	5	4	5
**Injection site 2 (no. examined)**	4	5	4	5
Inflammation, skeletal muscle, mononuclear cell	0	3 (1.3)	0	0
**Skeletal muscle**	4	5	4	5
Inflammation, mononuclear cell	0	3 (1.0)	0	1 (1.0)

Average severity (in parentheses) was calculated by adding all the grades of affected animals in the group and dividing by the number affected.

Table S20: Toxicology and Biodistribution stdies of replication deficient adenoviral vectors by intramuscular route

Vector (Ad species) origin	Transgene pathogen/antigen	Ad genomic deletions	Type of study	Species and strain	Vaccine dose	Vaccine regimen	Reference
GRAd32 (C) gorilla	SARS-CoV-2/Spike glycoprotein	ΔE1-ΔE3-ΔE4	SD Toxicity	Rabbit NZ	1×10^{11}vp	d1	this paper
			RD Toxicity	Rabbit NZ	1×10^{11} vp	d1-d15	
			Biodistribution	Rat SD	2×10^{10} vp	d1	
GRAd32 (C) gorilla	HIV/Polyepitope string	ΔE1-ΔE3	RD Toxicity	Rabbit NZ	1×10^{11} vp	d1-d21	this paper
			Biodistribution	Rat SD	3.3×10^{10} vp	d1	
ChAd155 (C) chimpanzee	Rabies virus/ G glycoprotein	ΔE1-ΔE4	Biodistribution	Rat SD	2.3×10^{10} vp	d1	*Napolitano F, PLOS NTD 2020*
ChAd155 (C) chimpanzee	Respiratory Syncytial virus/F-N-M2-1	ΔE1-ΔE4	RD Toxicity	Rabbit NZ	5×10^{10} vp	d1-d15-d29	*Stokes AH, IntJTox 2022*
			Biodistribution	Rat SD	1×10^{10} vp	d1	
			Shedding	Rat SD	1×10^{10} vp	d1	
ChAd3 (C) chimpanzee	Ebola/GP glycoprotein	ΔE1-ΔE4	RD Toxicity	Rabbit NZ	1.6×10^{11} vp	d1-d22	*Planty C, J.Appl.Tox 2020*
			Biodistribution	Rat SD	1.6×10^{10} vp	d1	
ChAdOx1 (E) chimpanzee	SARS-CoV-2/Spike glycoprotein	ΔE1-ΔE3	RD Toxicity	Mice CD-1	3.7×10^{10} vp	d1 –d22-d43	AZ COVID vax EMA/94907/2021
			Biodistribution	Mice CD-1	7×10^{9} vp	d1	*Stebbings R, Vaccine 2022*
AdC68 (E) chimpanzee	SARS-CoV-2/Spike glycoprotein	ΔE1-ΔE3	RD Toxicity	Rat SD/NHP Cyno	2×10^{11} vp /4×10^{11} vp	d1 - d15 (d19)	*Dai X, Immunotoxicology 2022*
			Biodistribution	Rat SD/NHP Cyno	2×10^{11} vp /4×10^{11} vp	d1	
Ad26 (D) human	SARS-CoV-2/Spike glycoprotein	ΔE1-ΔE3	RD Toxicity	Rabbit NZ	1×10^{11} vp	d1-d15-d29	J&J COVID vax EMA/158424/2021
Ad5 (C) human	SARS-CoV-2/Spike glycoprotein	ΔE1-ΔE3	Biodistribution	Mice BALB/c	1×10^{8} IFU	d1	*Dong Lee H, J.Microbiol.Biotechnol 2023*
several Ad5 (C) human	HIV/gag-pol, env Ebola or Marburg/GP glycoprotein	ΔE1-ΔE3 or ΔE1-ΔE3-ΔE4	RD Toxicity	Rabbit NZ	1-2 $\times 10^{11}$ vp/PU	d1 –d22 (d43)	*Sheets RL, J.Immunotox 2008*
Ad35 (B) human	HIV/env	ΔE1	Biodistribution	Rabbit NZ	0.5-1 $\times 10^{11}$ vp/PU	d1	
Ad5/35 chimera (C/B) human	HIV/gag	ΔE1-ΔE3	Biodistribution	Mice BALB/c	2×10^{9} vp	d1	*Shimada M, gene Therapy 2022*

Supplementary Table 20 Abbreviations: SD Tox= single dose; RD Tox=repeated dose; NZ=New Zealand; SD (referred to Rats)= Sprague Dawley; NHP=non-human primate; Cyno= Cynomolgus macaque
